# The penetration of financial capital and the growth of private hospital groups in Europe: the case of Spain (1975–2022)

**DOI:** 10.1017/mdh.2023.5

**Published:** 2022-10

**Authors:** Margarita Vilar-Rodríguez, Jerònia Pons-Pons

**Affiliations:** 1 Facultad de Economía y Empresa, University of A Coruña, Campus de Elviña, s/n, 15071 A Coruña, Spain; 2 Facultad de Ciencias Económicas y Empresariales, University of Seville, C/Ramón y Cajal, 1, 41018 Sevilla, Spain

**Keywords:** History, Health care, Hospital Groups, Spain, Twentieth and twenty-first centuries

## Abstract

From the last decades of the twentieth century, above all, in the more service-oriented post-industrial economies, and in a context of debilitation of public health systems, health care became exponentially profitable, thereby attracting new types of investors. In fact, this new stage entails moving from the commercialisation of health care to its financialisation; that is, medical care becomes just one more financial asset and its price and quality are quoted on the stock exchange. This study intends to participate in the debate initiated by historians of medicine and economic historians with the aim of tracing capitalist traits and market participation in the evolution of health coverage, a process initially promoted by professional doctors who converted their consulting rooms into small clinics and larger hospital companies and which, over time, saw the incorporation of financial capital. In particular, this paper has two specific objectives for the case of Spain. First, to analyse the relationship of collaboration and/or competition between public and private hospitals under democracy and the factors that have conditioned this relationship. Second, to make an initial contribution towards understanding how, in this context, the large private hospital groups have been created in Spain during this period, especially in recent decades with concentration in the hands of financial capital, originating from both the traditional banking sector and investment funds.

Hospital care is an essential part of the health system responsible for looking after one of human beings’ most precious assets: their health. The study of the historical development of hospital systems has become a subject of renewed interest in international academic circles due to its relevance to public health and the dependence on private and public hospital infrastructure for care provision.^1^ Private health insurance (PHI) has increased its weight within this system in recent decades, in a context of cutbacks, waves of privatisations, investment fund interest in this market and a crisis of the welfare state.^2^ As a result of this process, some European countries have a PHI market that supplements public coverage (e.g., Sweden, Spain, Ireland and the United Kingdom). This means that the private sector offers services already provided by the compulsory system, but with extra advantages such as shorter waiting lists and other benefits and comforts. In other countries, PHI plays a more important supplementary role by covering services or specialities excluded from the basic state package (e.g., Denmark, Hungary and the Netherlands). Finally, in some European Union (EU) member states, private insurance provides substitute cover for people excluded from certain aspects of the statutory health insurance scheme for various reasons, such as level of income or type of work (e.g., Germany). Overall, the causes behind PHI are very heterogeneous and a result of historical evolution, the power of different interest groups and the public policies implemented. Its increasing importance, however, is a common trend in all European countries.^3^ Bearing in mind this typology, the population covered by PHI in 2000 varied notably in Organisation for Economic Co-Operation and Development countries. Among the highest percentages, we find the case of the United States, where PHI covered 71.9% of the population (primary and supplementary), whereas in Europe, the case of the Netherlands stands out with 92% (28% as primary cover, and an additional 64% as supplementary). The lowest percentages correspond to Spain with 13% (2.7% primary and 10.3% duplicate or supplementary) and the United Kingdom with 10% (essentially duplicate or supplementary).^4^

Historically, depending on the time and place, the development of hospitals was based on a variety of charitable institutions, predominant before the nineteenth century. These coexisted, especially from this century onwards, with a constellation of establishments promoted by public and private activity, the market and formulas of solidarity of a civil nature, such as friendly societies.^5^ The coexistence or predominance of one typology or the other depended on both the historical framework and the idiosyncrasies of each country. The first studies available in the international field mainly focused on Northern European countries and the United States.^6^ Nevertheless, in recent decades, new contributions have enlarged the geographical scope of study, which has made it possible to obtain a more heterogeneous and global analysis perspective.^7^

Approaches from disciplines such as the history of medicine, social history and economic history have provided new qualitative and quantitative evidence that has served as a basis for conceptualising the diverse health coverage models and, within these, the different hospital systems in industrial societies. Comparison between countries is very complex due to the variety of institutions, the collaboration or competition between public and private institutions, the diversity of financing and management models that exist, the role of the state in the process, the degree of coverage of the population and so forth.^8^ Thus, in the decades preceding the Second World War, a coexistence of public and private charitable institutions funded by alms and taxes could be found, along with insurance mutuals created by civil associations or firms, PHI companies and limited public health coverage through the first compulsory social insurance schemes. This situation may be described as a mixed economy of welfare for the field of health and hospital care.^9^

During the mid-twentieth century, these diverse paths of hospital development became more integrated and regulated systems. The process was conditioned by various factors such as a country’s wealth, its traditions and its institutions,^10^ its political evolution and the conquest of social and political rights, the weight and financing capacity of the private and public sectors and the dissemination of medical and technological advances and medical professionalisation. By the end of the twentieth century, access to hospital in most EU countries was through different compulsory and universal medical insurance schemes within a broader system of social protection.^11^ However, private medical insurance has gradually gained weight in recent decades, above all since the ideological questioning of welfare states and the application of austerity policies with regard to social spending.^12^ In this respect, the outbreak of the crisis of 2008 had a decisive impact.^13^ As regards the United States, the gradual expansion of health coverage under the Social Security, through the well-known Medicare and Medicaid programmes, targeting the elderly population and low-income groups, respectively, has not overcome the inequalities in access to hospital care and has engendered a great political, business and social debate.^14^ Meanwhile, in countries with planned economy models, the market has been progressively introduced into the health economy since that late twentieth century. This is the case of China, a country where the encouragement of private enterprise after the death of Mao led to a growing commercialisation of hospitals and the erosion of the previous social protection systems, especially for rural populations.^15^

In general, the predominance of the World Bank over development policies in low-income countries, burdened by excessive debt, enabled the imposition of the ‘Washington Consensus’, which spread the idea that single-state welfare models were dysfunctional, while simultaneously recommending hospital provision through a variety of formulas with significant participation of the private sector and widely financed by paying users.^16^ These countries ended the twentieth century with a substantial foreign debt which conditioned everything, including the goal of universal health coverage, which was not even close to being achieved. A recent contribution on the development of the modern hospital examines the growth of this institution in the twentieth century, in different countries, taking into consideration its historical legacy. Focusing on the economic history of the hospital, the study outlines the forms of public and private provision and the political context in which the health systems were created. The collection provides a historical map of the world of different hospital models, including Spain, Brazil, Germany, Central and Eastern Europe, Great Britain, the United States^17^ and China. Overall, these comparative cases illuminate the complexities involved in each country and contribute new historical evidence to current debates on the organisation, financing and reform of health care.^18^

The prestigious academic journal *Bulletin of the History of Medicine* featured an interesting debate in 2020 between leading figures in economic history and the history of medicine.^19^ This collection of essays evidenced the need to combine knowledge from these fields in order to conduct a long-term analysis of such relevant questions as to what extent the logic of capitalism had influenced the functioning of markets, the making of profits and commercialisation throughout the history of medical care. In particular, understanding the capitalist system in its different stages is crucial to analysing the path that the health industry has followed in each country.^20^ From this perspective, we can ask ourselves how the capitalist institutions of medical care, including medical clinics, hospital companies and even insurance companies, have accumulated political influence and market power at different times, and what impact this different predominance has had on health coverage and on the welfare of the population. At the same time, it should be asked whether other legal formulas of health care provision such as friendly societies, cooperatives, trade union funds or lay and Church charitable entities may be considered as competitive capitalist threats for publicly organised medicine or as alternatives to the pursuit of profit in health care.^21^

In fact, the predominance of private actors in the provision of medical care and the behaviour of the prices of this provision reveal its market power and the strength of the state’s role in this area. It seems clear that, in the countries with solid public health systems, financed with social contributions or taxes, and with universal coverage, the private actors have had to seek market niches either to collaborate with public institutions of to cover market failures of the public coverage. It is also very interesting, in this respect, to study to what extent government-run health systems imitate or take advantage of the mechanisms of capitalist markets by, for example, using private organisations to administer public services or introducing competitive conditions in public health care schemes. On the contrary, when public health systems are marginal or are debilitated in terms of coverage and provisions, the market power of the private actors is enhanced and their field of action broadens. At this point, it should be asked to what extent modern health care markets are ‘embedded’ in state power or are a mixture of public and private power.^22^

In any case, there is no doubt that the advance and predominance of profit-seeking actions of hospitals, insurance companies and the pharmaceutical industry have contributed to a profound transformation in the functioning of the economy in general. At the same time, these changes have had consequences for the population’s health coverage and provisions and in terms of inequalities in health care.^23^ There is also no doubt that the economic crises of the 1970s marked a turning point in the management of hospital expenditure at a time when the welfare state and the role of the state in the economy were being called into question. With the argument of managing hospital resources in the most efficient way possible, the private sector assumed an increasingly important role in the main Western European countries. In this respect, it is necessary to extend research beyond the 1970s, when the flow of public and private money into medicine changed to include more powerful external actors such as large hospital corporations or venture capital funds.^24^ From the last decades of the twentieth century, above all, in the more service-oriented post-industrial economies, and in a context of debilitation of public health systems, health care became exponentially profitable, thereby attracting new types of investors ‘without special knowledge in the sector or interest in medicine itself’.^25^ This process has been described as ‘the destabilisation of medical care produced by a new type of monetisation’.^26^ In fact, this new stage entails moving from the commercialisation of health care to its financialisation; that is, medical care becomes just one more financial asset and its price and quality are quoted on the stock exchange.

That said, the question arises as to whether the health care ‘market’ may be considered as a market *per se* within the capitalist system.^27^ If it is, we should bear in mind the nature of the product. That is, among all the categories of goods and services, medicine and health care are among the most appreciated by the population, as they are crucial not only for people’s survival, but also for their welfare and quality of life.^28^ The recent global pandemic has only reinforced this basic idea. There is still much to be learnt about what causal relationships distinguish the economic history of health care. In dealing with this challenge, it is essential to establish the thread of historical continuity between the incipient capitalist forms, modern capitalism and the trend towards financialisation in recent decades, and how these changes have impacted on the provision of such an inelastic and fundamental good as the care of our health.

This paper aims to contribute to this international academic debate so relevant to the present moment, focusing on the case of Spain. Specifically, this paper aims to analyse the historical development of the private hospital sector in Spain coincident with the start of the democratic period, from 1975 to the present. The study intends to participate in the debate initiated by historians of medicine and economic historians with the aim of tracing capitalist traits and market participation in the evolution of health coverage, a process initially promoted by professional doctors who converted their consulting rooms into small clinics and larger hospital companies and which, over time, saw the incorporation of financial capital.^29^ In particular, this paper has two specific objectives. First, to analyse the relationship of collaboration and/or competition between public and private hospitals under democracy and the factors that have conditioned this relationship. Second, to make an initial contribution towards understanding how, in this context, the large private hospital groups have been created in Spain during this period, especially in recent decades with concentration in the hands of financial capital, originating from both the traditional banking sector and investment funds. The role of the hospital takes on special importance in the Spanish health system, which has been identified as ‘hospital-centric’ by some authors.^30^ The literature available for the case of Spain has highlighted the prominence of the hospital within the health system and has also determined defined stages in the development of public and private hospitals since the passage of the first compulsory sickness insurance in 1942.^31^ However, the historiography has also pointed out the existence of diverse territorial models in this area, especially since the devolution of health care responsibilities to Spain’s so-called Autonomous Communities (regions) after the establishment of the current democratic regime.

## Public and private hospitals in Spain since 1975: collaboration or competition?

In the twentieth century, the Spanish hospital system was largely determined by three fundamental laws: *Ley del Seguro Obligatorio de Enfermedad* [the law introducing compulsory sickness insurance], passed in 1942, *Ley de Bases de la Seguridad Social*) [the law establishing the Social Security], in 1963, and *Ley General de Sanidad* [a general health law; hereinafter LGS] in 1986.^32^ The first two contained the dictatorship’s political strategy in the hospital sphere, which lead to delaying the creation of a modern, coordinated hospital system in Spain until the 1970s. The LGS, passed during the democratic stage that was initiated in Spain in 1978, incorporated two key aspects for hospital interests. First, the strictly public management model contemplated *a priori* in the LGS was soon called into question as the private sector was guaranteed a portfolio of ‘privileged’ clients: civil servants and some other public employees. Thus, the law retained the possibility of an annual choice of insurance for the mutual funds of these public servants, a possibility that constituted an exception within the national health care model.^33^ In 1989, of the six million people covered by private companies, around two million corresponded to public servants who had opted for the private sector instead of social security, whereas the rest had double coverage (public and private).^34^

Second, the LGS incorporated the possibility of ‘establishing agreements for the provision of health care services with means external’ to the public health administrations, which meant the possibility of agreements with private health care and also considered the option of private hospitals linked to the public health service and patients treated in authorised private centres at the expense of the public administration. In fact, these first two aspects were already being applied within the health framework in Spain and the LGS only consolidated them. It should be borne in mind that the mutual funds of public servants had been functioning since the 1970s, and public expenditure on agreements with private health services had not ceased to grow since the start of Spain’s democracy.^35^ In general, from the times of the Franco dictatorship, the decision had been taken to develop an independent health care network for the Spanish health system rather than reaching agreements with existing service providers in the public sphere, such as municipal and provincial hospitals.^36^ However, and in parallel, a policy of special agreements was consolidated, which led the public health sector to a clear financial and consumer dependence on private health care. In this way, the demand for public health care became a key source of profits for private health businesses. This process can be seen clearly in the hospital sphere, and it was consolidated to a certain extent by the LGS of 1986.

Third, the belated passage of the LGS meant that this process overlapped with the devolution of responsibility for health care to the autonomous communities.^37^ Consequently, the law afforded regional governments considerable scope to manoeuvre and only established the general foundations of the health system and guarantees to maintain a certain coordination of the system as a whole. This situation led to the consolidation of different territorial models of health management, characterised by significant inequalities in the public–private hospital tandem, in terms of both management and the provision of services, which had already become evident before 1986.^38^

The years following the passage of the LGS were notable for two processes. First, public health coverage in Spain reached 81% of the population in 1975, 90% in 1985 and 99% in 1990, after the inclusion of the population treated up to then by charitable services.^39^ Second, the LGS initially set up a mixed funding structure for the national health service, with most funds coming from income through social contributions. Nevertheless, the *Ley de Presupuestos Generales del Estado* [the law regulating the general state budget] passed in 1989 modified this system of financing health care by introducing taxes as the main source of funding. Within this process, the weight of social contributions was gradually reduced in favour of state funding through taxes between 1980 (75.2% and 24.8%) and 1989 (27.25% and 72.8%). A decade later, in 1999, the first budget that included complete funding of the public health system on the basis of taxes was passed.^40^

A key element in this situation was the tax reform promoted by Fernández-Ordóñez in 1977, as this increased the tax-raising capacity of the Treasury. The fiscal reform introduced the taxation principles of the welfare state and established some taxes equivalent to the rest of the EU.^41^ In practice, two key elements can be highlighted. On the one hand, the number of taxpayers increased because personal tax privileges, which some employers and professionals benefitted from, ceased to exist. This was true, above all, for capital income, due to the disappearance of bank secrecy. On the other hand, tax revenue increased to new levels that would have been impossible with the liberal tax system, with the raising of legal tax bases and the obligation to file tax declarations. It was quite another matter, however, whether fraud would actually disappear under democracy and whether in practice the tax system would really be as progressive as in the legislation.^42^

Within this context, the configuration of the public and private hospital system barely experienced changes from 1986, except a slight trend towards a percentage reduction in the number of private hospitals and an increase in their size in terms of beds (Table 1). This was basically due to takeover and merger processes in this area in order to gain investment capacity and modernise facilities during a stage of great technological changes and expansion of the public hospital system.

**Table 1.** Composition of the public and private hospital system in Spain

Proprietorship	Number of hospitals					
	1970	1986	1996	2005	2010	2019
% public	37	42	46	46	46	47
% private	63	58	54	54	54	53
Total Spain	1 408	924	855	849	878	879

	Number of beds					
Proprietorship	1970	1986	1996	2005	2010	2019
% public	68	69	69	67	67	67
% private	32	31	31	33	33	33
Total Spain	169 841	193 171	167 429	157 808	161 188	159 175

Source: Prepared by the authors on the basis of the *Catálogos Nacionales de Hospitales*, 1970, 1986, 1996, 2005, 2010 and 2019.

If one analyses the internal composition of the proprietorship of private hospitals from 1970, a fall in the number of hospitals of around 30% for each type can be observed (Table 2). In this section, private charity hospitals show the slightest fall in the number of hospitals. It must be taken into account that this category includes the so-called third sector hospitals, that is, hospitals belonging to the Church and private foundations, which have experienced a notable growth in recent decades. With regard to the number of beds, the evolution is very similar, a fall in all typologies and slighter in private charity hospitals. Moreover, it is important to note that although the number of for-profit private hospitals has fallen (1970: 682 and 2019: 317), the number of beds they provide has increased (1970: 26 011 and 2019: 29 434). This once again reaffirms the increase in the size and capacity of these facilities in recent decades. This clearly indicates that it was the small clinics and hospital companies that gave way over time, either because they closed due to being unable to compete with large companies and private health enterprises, or because they were taken over by these larger concerns.

**Table 2.** Private hospitals classified by proprietorship

	1970	1985	1996	2005	2010	2019
Number of hospitals						
Private Charity	77	52	61	61	60	64
Spanish Red Cross	36	32	18	12	12	10
Church	90	69	62	60	60	60
MATEP	–	–	23	21	17	17
Private	682	383	299	308	323	317
Total NH private sector	885	536	463	462	472	468
Total NH public and private	1 408	924	855	849	878	879
Number of beds						
Private Charity	8 762	9 225	7 772	7 982	7 978	8 265
Red Cross	2 359	3 492	2 146	1 534	1 620	1 125
Church	16 950	14 358	12 179	11 895	11 883	11 873
MATEP	–	–	1 741	1 595	1 237	1 072
Private	26 011	32 848	28 413	28 708	30 984	29 434
Total NB private sector	54 082	59 923	52 251	51 714	53 702	51 769
Total NB public and private	169 841	193 171	167 429	157 808	161 188	159 175

Abbreviations: NB, number of beds; NH, number of hospitals; MATEP, mutuas de accidentes de trabajo y enfermedades profesionales (workplace accident and occupational illness mutuals).

Source: Prepared by the authors on the basis of the *Catálogos Nacionales de Hospitales*, 1986, 1996, 2005, 2010, 2015 and 2019.

As well as this process, it is also possible to observe a certain progressive specialisation of private hospitals during the period under study (Table 3). Hence, Church-owned hospitals have become predominant among psychiatric and children’s hospitals, whereas the hospitals of private companies have been concentrated above all as general hospitals and also as surgical hospitals. It is noteworthy that private charity hospitals have gradually and progressively lost weight over time, except in the ‘Geriatrics and long stay’ category, which is also an area of growing interest for private companies in a context of progressive ageing of the population and the possibility of signing agreements with public institutions for the subsidised provision of hospital beds. However, as already mentioned above, the so-called third sector hospitals, that is, those belonging to private foundations, are included within the ‘private charity hospitals’ category. This typology and its functioning need to be examined in greater depth in future versions of this study.

**Table 3.** Composition of private sector hospital beds in Spain by typology

Typology	1986	2019
*1. General hospitals*	24 975	29 435
Private (%)	60	66
Church (%)	7	15
Private Charity (%)	24	16
Spanish Red Cross (%)	9	3
*2. Surgical hospitals*	14 398	1 554
Private (%)	81	69
Church (%)	14	25
Private Charity (%)	5	5
Red Cross (%)		1
*3. Children’s hospitals*	1 172	491
Private (%)	36	14
Church (%)	63	82
Private Charity (%)	1	4
*4. Psychiatric hospitals*	13 211	8 875
Private (%)	33	36
Church (%)	63	60
Private Charity (%)	4	4
*5. Geriatrics and long stay*	–	7 956
Private	n.d.	54
Church	n.d.	12
Private Charity	n.d.	33
Red Cross	n.d.	1
*6. Others*	6 167	3 458
Total private sector beds	59 923	51 769

Source: Prepared by the authors on the basis of the *Catálogos Nacionales de Hospitales*, 1986 and 2019.

In this respect, it must be borne in mind that after the passage of the LGS and the devolution of health care responsibility to the autonomous communities, there was an increase in the number of agreements signed with private hospitals. Thus, from the 1990s, around 60% of the beds of private hospitals were included in agreements to provide public health services in exchange for state subsidies in order to treat patients referred from the public health system (Table 4). This evolution evidences the important weight that the agreements with the public health sector have had for the private health care business, which has resulted in a relationship that is more of cooperation than of competition. Overall, almost 30% of hospitals and 20% of the total number of beds available in the hospital sector in Spain over the last 30 years have corresponded to private hospitals authorised and subsidised to provide public health services.

**Table 4.** Private sector hospitals and beds authorised and subsidised to collaborate with the public health system in Spain by proprietorship

Proprietorship	Number of hospitals			Number of beds		
	1996	2005	2019	1996	2005	2019
Spanish Red Cross	12	6	7	1 307	639	710
Church	33	40	41	6 681	8 236	8 551
MATEP	5	5	4	501	506	200
Private Charity	51	49	34	6 847	6 948	5 613
Private Non-Charity	119	152	147	16 727	18 644	15 749
Total private	220	252	233	32 063	34 973	30 823
Total private sector	463	462	468	52 251	51 714	51 769
Total Spain	855	849	879	167 429	157 808	159 175
State-subsidised^a^ % of the private sector	48	55	50	61	68	60
State-subsidised as % of total in Spain	26	30	27	19	22	19

Abbreviation: MATEP, mutuas de accidentes de trabajo y enfermedades profesionales.

a Private hospitals subsidised by the state to provide public health care services.

Source: Prepared by the authors on the basis of the *Catálogos Nacionales de Hospitales*, 1996, 2005 and 2019.

Therefore, although the LGS enshrined, as a general rule, direct state management with the public administration’s own staff and resources, the actual situation turned out to be less clear-cut. The legal coverage of this situation was clarified with the three exceptions to this direct management established in article 90 of the LGS. First, it should be noted that the supply of most medicines was through private pharmacies. Second, there were the beneficiaries of the aforementioned mutual funds for public servants that financed health care (Muface, Isfas and Mugeju). These mutuals had not developed their own infrastructure and hired services from private and public companies, then beneficiaries could choose from these provided that they had a current agreement with the national health service. Finally, the LGS established the possibility of signing agreements with the private sector for the provision of services.^43^ This philosophy of collaboration between public and private sectors in the area of health care in general and with regard to hospitals in particular was consolidated with the passage of Law 15/97, of 25 April, on enabling new forms of management in the national health system.^44^

## The creation of the main private hospital groups in Spain

The modification of the system of funding health care introduced in the *Ley de Presupuestos Generales del Estado* for 1989 led, as already mentioned above, to a progressive increase of state contributions to the financing of social security. This increasing state financing via taxes was allocated not only to funding directly managed public institutions, but also to funding private institutions such as employers’ industrial accident mutuals, the mutual funds of public employees or other private health care companies, and including hospitals that had signed the corresponding agreements. The continuance of these hospital agreements reveals the interest of private insurance companies and their hospitals in participating in the distribution of public expenditure, which was to become an essential part of their business. The spirit of participation in the process of obtaining this public money can be found in the numerous reports and declarations of the *Instituto para el Desarrollo e Integración de la Sanidad* [Institute for the Development and Integration of Health Care; hereinafter IDIS], which classify this source of income as ‘necessary collaboration’.^45^

At their peak, the agreements signed with the mutual funds of civil servants and public employees led to 94% of these public servants being covered by private insurance.^46^ This high number of policies favoured the expansion of PHI companies, boosting the figures of their business. With this guaranteed demand, health insurance companies started to reorganise the branch. The first step involved the reduction of the large number of companies operating in this line. The total number of companies active in the health care branch fell from 243 in 1984 to 132 in 1990.^47^ This initial process of concentration was based on a strategy of reducing the number of companies in the sector by means of mergers involving local groups, *igualatorios* (doctors’ associations) and companies founded by doctors in almost all provincial capitals, which gave rise to stock companies operating at national level.

Most for-profit private hospitals were founded by insurance companies or doctors’ associations who agreed the provision of services with the insurers. Thus, for example, one of the leading health insurers today – Sanitas – had preferential agreements with Organización CEYDE, S.A. in the 1950s. Later, in the 1970s and 1980s, many insurance companies built or renovated clinics to create their own network. This was the case of Igualatorio Médico Quirúrgico, S.A. in Bilbao, which acquired Clínica Vicente in San Sebastián in 1980, or Asistencia Sanitaria Colegial, S.A., which purchased a hotel in Barcelona in 1989 and converted it into Hospital de Barcelona. In this way, the number of hospital infrastructures owned by insurance companies was increasing, and these hospitals treated private patients and members of mutuals and supplemented this with agreements with the public authorities. Since the 1990s, changes in PHI companies have been conditioned by various factors, two of which are particularly notable. On the one hand, the devolution of responsibility for health care to the autonomous communities. On the other hand, the interest of the national banking sector and international investment funds in Spanish private insurance companies and private hospitals. These two elements have led to a segregation of the hospital business and the creation of large hospital groups in Spain. From the point of view of those running private clinics, the sale of small clinics and the creation of large groups is explained by rising health care costs.^48^

The strategy of introducing new models of hospital management had an important role in the consolidation of the hospital groups. These consisted in separating health care provision from financing.^49^ The model was disseminated in the following years, and by 2000, more than seventy public health bodies were managed privately in Spain. The participation of the private health sector in the management of public health care through different legal forms even reached the point of including total management. Some private hospitals were created with the main aim of signing an agreement with the public health authorities and providing coverage to the beneficiaries of the Social Security.^50^ Galicia was one of the first regions to implement the above changes, followed by Catalonia, Valencia and Madrid under the aegis of conservative governments. These business opportunities stimulated the creation of hospital groups such as Capio, Ribera Salud and USP-Quirón.

Overall, at the end of the twentieth century, the financial needs arising from technological changes in the area of health care, and the opportunities provided by institutional changes and new political strategies, favoured merger processes and the entry of national banks and international investment funds into the private hospital sector. This complex process led to the emergence of the five large hospital groups that occupy the leading positions in the sector ranking in Spain today: Quirón Salud, Vithas, HM Hospitales, Ribera Salud and HLA.^51^ These five private groups account for 82% of turnover in the sector. This is a very relevant figure if one takes into account that the private non-charitable hospital market in Spain moved 6 775 million euros in 2020 (Table 5). A total of 64% of this income came from agreements signed with insurers, 26% from agreements signed with the public sector and only 10% corresponded to purely private expenditure coming from out-of-pocket payments by patients.^52^

**Table 5.** Estimated turnover of the main hospital groups 2020 (millions of euros)

	Managing group of company	Headquarters	2020	%
1	Quirónsalud Group^a^	Madrid	3 475	51.29
2	Vithas Sanidad, S.L. (Group)	Madrid	567	8.37
3	HLA Lavinia Salud, S.L. (HLA Hospital Group)	Madrid	466	6.88
4	HM Hospitales (Group^b^)	Madrid	435	6.42
5	Ribera Salud, S.A. (Group)	València	410	6.05
6	Hospiten Holding, S.A. (Group^a^ ^,^^b^)	Santa Cruz de Tenerife	330	4.87
7	Sanitas, S.A. de Hospitales	Madrid	284	4.19
8	Clínica Universidad De Navarra	Pamplona/Iruña	227	3.35
	Estimated total turnover of the sector		6 775	

Note: The data have been obtained from the Institute for the Development and Integration of Health Care (IDIS), 2021, which warns that its source only provides data for non-charitable private clinics. For this reason, it does not include the turnover of not-for-profit hospital groups such as those belonging to the Church: *San Juan de Dios* [Saint John of God] or *Hermanas Hospitalarias* [Sisters Hospitallers].

a Includes income arising from the management of hospitals abroad.

b Includes estimate from DBK.

Source: IDIS, Análisis de la situación de la Sanidad Privada en 2021; available at https://www.fundacionidis.com/informes/analisis-de-situacion-de-la-sanidad-privada/sanidad-privada-aportando-valor-analisis-de-situacion-2021.

It should be pointed out, however, that there are a number of large hospital groups that are excluded from this classification because they are owned by the Church or another kind of private charity in the form of foundations. If the ranking by number of hospital beds is considered, one can see that first position is occupied by the *Orden Hospitalaria de San Juan de Dios* [Brothers Hospitallers of Saint John of God] with thirty-three hospitals operational and a total of 8 312 beds, and the *Hermanas Hospitalarias del Sagrado Corazón de Jesús* [Sisters Hospitallers of the Sacred Heart of Jesus] are in third place with fifteen hospitals and 4 591 beds. There is little information available on the turnover of Church hospitals (Table 5).^53^ Through religious orders and movements, the Church controlled more than fifty hospitals in Spain in 2020, with around twelve thousand beds, which accounted for 7.3% of the total amount. This hospital network has a long history (Tables 3 and 4). Although in theory these hospitals compete with private companies, in most cases, they are identified as non-profit making entities, which gives them important tax advantages. Despite this condition, they generate significant income, partly deriving from agreements signed with the public authorities and insurance companies.^54^ This situation has generated a certain amount of unrest among business groups in the sector who question whether, for Church hospitals ‘with private ownership, enormous revenue and a national health system that treats the entire population, it is logical that they continue to enjoy special conditions with respect to the competition’.^55^

Overall, in 2019, thirteen hospital groups concentrated 43% of private hospitals in Spain and 55% of the beds of the private hospital sector (Table 6). The trend towards concentration and increasingly large companies in this area seems clear, which gives these large groups greater market power and greater capacity to exert pressure when negotiating with the public health system.

**Table 6.** Private hospital groups by market share of hospitals and beds in Spain

Group	2010		2012		2014		2016		2018		2019^a^	
	A	B	A	B	A	B	A	B	A	B	A	B
Quirón	1.5	1.9	4.1	4.1			9.5	12.3	10.0	13.2	10.0	13.0
San Juan de Dios	5.8	12.8	6.5	12.0	6.4	12.1	6.6	12.2	5.9	11.4	8.0	15.0
Vithas			2.2	2.0	2.2	2.0	2.7	2.3	4.1	4.0	5.0	5.0
HM Hospitales^b^	1.0	1.1	1.1	1.2	1.1	1.2	2.4	2.1	3.3	3.1	3.0	3.0
HLA^c^							2.9	2.4	3.0	2.4	3.0	2.0
Hermanas Hospitalarias	3.1	10.2	3.7	8.3	3.7	8.5	3.5	8.2	2.6	7.4	3.0	8.0
Hestia Aliance							2.0	3.2	2.0	3.1	3.0	4.0
Viamed							2.2	1.4	2.2	1.4	3.0	2.0
Hospitales Católicos de Madrid							2.0	2.8	2.0	2.7	2.0	2.0
Hospiten	1.9	1.7	1.7	1.7	1.8	1.6	1.5	1.7	1.5	1.7	2.0	2.0
Grupo Hospitalario Recoletas	1.9	1.3	1.7	1.3	1.5	1.0	1.5	1.0	1.5	0.8	2.0	1.0
José Manuel Pascual Pascual	1.2	2.4	1.3	2.2	1.3	2.2	1.1	2.2	1.5	2.4	2.0	2.0
Cruz Roja (Red Cross)	1.2	1.0	2.0	2.4	2.0	2.4	2.2	2.0	2.2	2.2	1.0	1.0
Hospitales Nisa	1.5	2.2	1.5	2.2	1.5	2.2	1.5	2.3				
Red Asistencial Juaneda							1.1	1.1				
Sanitas			0.7	0.5	0.7	0.5	0.9	0.7				
Asisa			2.8	2.3	3.1	2.4						
GHQ					4.6	5.2						
IDC Salud					2.9	4.5						
Capio	2.9	2.9	2.0	3.4								
USP	2.5	2.4										
Ruber	0.4	0.6										
Rest	76.8	59.6			66.2	62.2	52	37.5	58.3	44.2	53	40
TOTAL^d^	100	100	100	100	100	100	100	100	100	100	100	100

Note: A: market share hospitals (%); B: market share beds (%).

a The information referring to 2019 is provided in the reports for both 2020 and 2021, and this last source has been used due to being more up to date.

b HM, Hospital de Madrid, S.A. constituted on 23 December 1989. Archivo del Registro Mercantil de Madrid, inscripción 1, tomo 134, fs. 149-60, June 1990.

c HLA LAVINIA SALUD S.L. constituted in June 2015 by Asisa insurance company as sole proprietor. Archivo del Registro mercantil de Madrid, inscripción 1, tomo 33688, M-606437, fs. 80-4.

d The sum of the columns does not add up exactly to 100. This is due to the rounding of decimals used in the source we have utilised.

Source: Análisis de Situación de la Sanidad Privada. IDIS report for the years 2021, 2019, 2018, 2017, 2016, 2015, 2014, 2013 and 2011.

As a whole, the private hospital sector in Spain is characterised by the presence of a number of actors that may be classified in three large groups: hospital groups, hospitals belonging to health insurance companies and independent hospitals. The IDIS report shows that the hospital groups account for 51.4% of private hospitals and 64.3% of private beds available in Spain.^56^ The insurance companies, for their part, have 3.3% of private hospitals and 3.5% of beds. Meanwhile, independent hospitals and clinics account for 45.3% of private hospitals and 32.2% of beds (Table 7).

**Table 7.** Distribution of private hospitals and beds in accordance with the main actors (in percentages)

Year	Hospital groups		Insurance companies		Independent hospitals	
	Market share hospitals	Market share beds	Market share hospitals	Market share beds	Market share hospitals	Market share beds
2010	30.0	44.0	8.0	8.0	62.0	48.0
2011	26.2	36.2	5.5	5.4	68.3	58.4
2012	31.0	42.0	4.0	3.0	65.0	55.0
2013	32.0	44.0	4.0	4.0	64.0	52.0
2014	37.0	50.0	4.0	4.0	58.0	46.0
2015	38.0	51.0	5.0	5.0	57.0	44.0
2016	45.0	58.0	2.0	3.0	53.0	39.0
2017	46.0	61.0	2.0	3.0	52.0	36.0
2018	48.0	62.0	3.0	3.0	49.0	35.0
2019	48.0	61.6	3.1	3.5	48.9	34.9
2020	51.0	66.0	3.2	4.0	45.8	30.0

Source: *Análisis de Situación de la Sanidad Privada. Informe IDIS,* 2011, 2012, 2013, 2014, 2015, 2016, 2017, 2018, 2019 and 2021.

Within this map, over the last two decades, there has been a clear process of concentration of private hospitals by large hospital groups that control the ownership of an increasing number of hospitals. Their market share in terms of both number of hospitals and number of beds grew by twenty points between 2010 and 2020.

In order to understand the formation of these groups in the long term, we shall focus on the historical creation of two leading groups: Quirón and Vithas. This process is complex in the case of the Quirónsalud group, which has undergone enormous changes, especially since 2012 (Table 8).^57^ The origins of the company date back to the foundation of Igualatorio Médico-Quirúrgico y de Especialidades in 1932 in Zaragoza, made up of a group of doctors with coverage limited to the local area. In 1955, Publio Cordón purchased the company, which then became Previsión Sanitaria, S.A. The company’s first clinic was opened in 1957. An important landmark for its growth was the agreement with Muface, which at the beginning of 1977 obliged the company to operate throughout the whole country. The insurer was in the hands of the Cordón family until its sale to the DKV group in 1998, which disposed of the health services provision business and just kept the Quirón hospital group.^58^ Its rapid growth soon attracted venture capital funds such as the multinational Doughty Hanson, which entered the company’s capital and, in 2012, merged the company with USP Hospitales.

**Table 8.** Historical transformations of the IDCQ Hospitales y Sanidad S.L. group (QUIRÓN)

2016: IDCQ Hospitales y Sanidad S.L. (Group acquired by German company Helios). Belongs to FRESENIUS.	2014: Quirón. Hospital Group merger with IDC Salud – QUIRÓN.	2013: Change of name for the old name of IDC Salud	2011: Capio sells to the CVC venture capital fund again.	2006: Capio Sanidad sell part to Apax venture capital. (At this time, it has twenty hospitals in Spain.)	2005: CVC sells IDC to Swedish group Capio.	1998: CVC acquires Ibérica de Diagnóstico y Cirugía (IDC). 2002: Ibérica de Diagnóstico y Cirugía (IDC). Acquisition of Jiménez Díaz Foundation.
		2012: USP – Quirón Hospital Group merger.	USP: European Division of United Surgical Partners International. Property of Doughty Hanson venture capital.	2003: Hospital de Marbella (Málaga).	2000: Hospital San Camilo (Madrid). 2001: Hospital San José (Madrid) and Hospital San Carlos (Murcia).	1998: Purchase of: - Instituto Universitario Dexeus (Barcelona). - Clínica La Esperanza (Vitoria). - Hospital Santa Teresa (La Coruña). - Clínica Sagrado Corazón (Seville).
			Quirón	1998: Sale of Previasa. Quirón Hospitales, S.L. keeps the hospital companies.	Publio Cordón family purchase. Previasa 1955.	Igualatorio Médico Quirúrgico y de Especialidades. Zaragoza (1932).

Source: Prepared by the authors on the basis of data from the *Archivo del Registro Mercantil de Madrid* and press news articles.

USP Hospitales had been formed in Spain in 1998 as a European division of United Surgical Partners International under Gabriel Mas Furroll, its president and managing director, and acted as a private and independent hospital group until the start of 2010. A month and a half before the merger with Quirón, Doughty Hanson had acquired USP Hospitales from Barclays and the Royal Bank of Scotland for 355 million euros. These banks had taken control of USP when CINVEN, its previous owner, was unable to meet its debts. This transaction was completed by Doughty Hanson & Co alone, before the merger with the Quirón group, in order to avoid problems with the Competition Commission.^59^

In 2014, the investment fund CVC, owner of IDC Salud, got involved and replaced Doughty Hanson as the leading shareholder of Quirón. In June 2014, a merger agreement was reached between IDC Salud (previously owned by the Swedish group Capio) and the Quirón hospital group, which gave rise to the Quirónsalud hospital group.^60^ Ibérica de Diagnóstico y Cirugía (IDC) had been created in the mid-1990s, with the doctor and entrepreneur Víctor Madera at the helm. It was acquired by the powerful investment fund CVC in 1998, although Madera continued running the company and based his growth strategy on the outsourcing of public health services.^61^ In 2002, it became the leading shareholder of the historical Jiménez Díaz Foundation in Madrid. In 2005, CVC sold IDC, which had now become the leading private health manager in Spain by turnover, to the Swedish group for three hundred million euros.^62^ One year later, the American venture capital fund Apax took over Capio for two thousand million euros. In 2011, CVC bought back the Spanish unit of Capio for nine hundred million euros, went back to using the name IDC and maintained Víctor Madera in charge of the management of the hospital group.^63^

Within this framework, in 2015, an important business concentration operation took place with the registration in the *registro mercantil* [registrar of companies] of a proposed merger by takeover, with Quirón Hospitales, S.L. as the acquiring company and seventeen companies domiciled in Madrid, Barcelona, Zaragoza, Bilbao and Vitoria as the companies being acquired.^64^ The result was the foundation of IDCQ Hospitales y Sanidad S.L., which started operating on 26 June 2015.^65^ In February 2021, Idcsalud Holding, S.L.U. (*Sociedad de Responsabilidad Limitada Unipersonal*; that is, a single-member private limited liability company or SUP) was acquired by Helios Healthcare Spain, S.L.U. and, then, this company became the sole shareholder of IDCQ Hospitales y Sanidad S.L.U.^66^ It should also be pointed out that the main shareholder of the Fresenius group is owned by a non-profit foundation called Else Kröner-Fresenius-Stiftung, whose main objective is the development of medical and humanitarian projects.^67^

Vithas, for its part, second in the ranking by turnover in 2021, has followed a similar process. The historical creation of this group has been determined by the final union of three business lines: the hospitals of Adeslas insurance company, acquired by Caixabank in 2009; the Goodgrower group, an investment group created in 2008 by the Gallardo family (founder and controlling shareholder of the Catalan pharmaceutical group Almirall) to invest in the health sector and the NISA group, acquired in 2017.^68^ The process started in 1966, when Unión Médica Regional, S.A. was founded in Granada as a health insurance company. The nineteen founding partners were all doctors, except for an insurance agent and a lawyer. The company was created with a capital of one million pesetas distributed in fifty-five shares per partner, except for the lawyer who only acquired ten.^69^ Adeslas became a shareholder in 1986, and went on to become the main shareholder by acquiring its entire portfolio in 1991,^70^ when its name was changed to UMR, S.A. In 2003, a capital increase proposed by Adeslas was accepted and it now became UMR, S.L., a holding of Adeslas, which acted as manager of the hospital interests of the parent company.^71^

When SegurCaixa, belonging to the bank holding company Caixabank, acquired Adeslas in 2009, it chose to separate and dispose of its hospital assets. Adeslas had participated in the concentration process in this branch through the acquisition of local companies from 1991. This path had coincided with the entry of foreign capital into its shareholding, when the French group Méderic acquired 45% of its capital.

However, when VidaCaixa Adeslas sold its non-life insurance business to Mutua Madrileña for 1 075 million euros, also in 2009, it excluded a group of ten hospitals that it put up for sale separately (Table 9). These hospitals together had around one thousand beds, and, in 2009, they were being used by over one and a half million patients. These hospitals then went on to depend on a subsidiary wholly controlled by Criteria. What the sale to Mutua Madrileña did involve included sixty-five dental clinics, thirty-two medical centres and the public–private concession for the use of Hospital de La Ribera in Alzira.^72^

**Table 9.** Historical transformations of the VITHAS group

2021: Vithas Sanidad S.L.	2017: Vithas buys NISA.	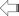 2012: Caixabank, S.A. sells 80% to Goodgrower. 2012: Change of name to Vithas Sanidad, S.L.	2003–4: New name UMR, S.L. proposed by Adeslas. Management holding of hospital companies.	1998: Change of registered office to Madrid.	1991: Sold the entire portfolio to Adeslas.	1966: Unión Médica Regional S.A. Granada (Doctors).
		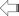 2011: Goodgrower (Gallardo family buys 80% of the hospitals from Criteria).	2009: Adeslas acquired by Segurcaixa. Separation of hospital group in hands of Criteria.	Adeslas: made up of ten hospitals.^a^		
		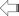 2007: NISA with seven hospitals in Spain providing 1 100 beds in Valencia, Alzira, Castellón, Madrid and Castilleja de la Cuesta.		1998: Change of name to Nuevas Inversiones en Servicios, S.A. (NISA).	Acquisition of numerous hospitals.	1970: Clínica Virgen del Consuelo (Valencia).

a The hospitals were: Ntra. Sra. de Fátima (Vigo), Santa Catalina (Las Palmas), Ntra. Sra. La Salud (Granada), Nra. Sra. de América (Madrid), Virgen del Mar (Almería), Perpetuo Socorro (Alicante), Parque San Antonio (Málaga), Montserrat (Lleida), Santa Cruz (Tenerife) and San José (Vitoria).

Source: Archivo del Registro Mercantil de Madrid and press articles.

In 2012 UMR, S.L. changed its business name to Vithas Sanidad S.L. due to its acquisition by the Gallardo family (Goodgrower), which in turn had acquired the hospitals of Adeslas after its takeover by SegurCaixa.^73^ The investment company Goodgrower had acquired 80% of the hospitals from La Caixa in 2011, which kept 20% within its Criteria CaixaHolding, and relaunched the hospital group with this new brand.^74^ Later, in 2017, Vithas acquired 100% of the Valencian company NISA,^75^ of which it already owned 45%.^76^ The origins of the NISA group date back to 1967, when a group of doctors formed the company Clínica Virgen del Consuelo S.A. in Valencia, inspired by the lack of private health care facilities in the city. It started operating in 1970. In 1991, the company acquired Hospital San Juan de Dios, which was renamed as Hospital Nisa Valencia al Mar. In 1998, the name of Clínica Virgen del Consuelo S.A. was changed for the present name NISA, an acronym for Nuevas Inversiones en Servicios, S.A. In 1993, Hospital Nisa 9 October was inaugurated, also in Valencia. In 1998, Hospital Nisa Aguas Vivas was incorporated into the network. In 2003, Hospital Nisa Rey Don Jaime was inaugurated in Castellón de la Plana.^77^ In 2007, Hospital Nisa Sevilla-Aljarafe, in Castilleja de la Cuesta,^78^ and Hospital Nisa Pardo de Aravaca, in the Aravaca neighbourhood in Madrid, were opened to the public.^79^ In 2010, Nisa inaugurated the Centro de Daño Cerebral Nisa Vinalopó, a drain damage centre linked to Hospital Nisa Aguas Vivas, and, in 2011, Centro Médico Nisa San Bernardo was opened in the centre of Seville. In January 2017, 100% of Hospitales Nisa S.A. was purchased by the Vithas Group.^80^

This purchase transaction reinforced the Vithas Group’s position as the second group in the sector and enabled it to earn an overall income in excess of five hundred million euros. With a significant presence, above all, in Madrid and Valencia, it has nineteen hospitals, twenty-five specialised centres and almost 6 800 employees.^81^ After these years of expansion, the Gallardo family bought the part held by CriteriaCaixa in 2021, now having full ownership of the entire hospital group.^82^

## Conclusions

Private health care in general has experienced an increase in activity in Europe, especially in its role as a supplementary provider of public health coverage in recent decades. This paper has shown that, as a result of this process, modern health care markets are a mixture of public and private power. Within this framework, the weight of private hospital groups has also been on the rise. On the demand side, the factors that have driven this process have been the reduction in public spending, the privatisation of public health services, tax incentives for private insurance and changes in the population’s consumption patterns. On the supply side, there has been growing interest in the health care industry among the banking sector, investment funds and general insurance companies.

As regards the case of Spain, the hospital companies have consolidated thanks to the demand generated by private insurance companies to cover both their private patients and those linked to the different mutual funds for public servants, the agreements with the Social Security and the public health institutions created by the autonomous communities, and also thanks to the management of publicly owned hospitals through foundations, from which they obtain enormous profits without direct public control. Other data should be taken into account, such as the percentage of public health expenditure that is allocated to the agreements with the private sector. The private hospital and health care groups, therefore, generate their business thanks to private demand, but they participate in public demand, in which they aspire to grow. In 2017, agreements with private health centres accounted for 11.2% of total public expenditure on health.^83^ The lobbies of the sector, such as IDIS in Castellón de la Plana,^84^ defend the virtues of this collaboration in all private and public circles.^85^

This situation has attracted national and international capital (investment funds, the banking sector and insurance companies), which, with a clearly speculative objective, is investing in the growing health sector, buying and selling its shares in hospital companies with the aim of making large profits in the short term.^86^ Independent hospitals are losing ground in the market of health coverage provision, whereas the large groups are gaining weight, concentrating hospitals and beds and modifying the historical tradition of dispersion and independence of local and regional companies in the hands of doctors from the area, creating a monopoly controlled by financial capital. Moreover, these groups are also diversifying into other lines of business such as care homes for the elderly and dental clinics, other highly profitable niches of the health care business.

These changes raise numerous questions on the future of the sector. What will be the place of medical and health criteria when these groups need to take strategic decisions? What will their contribution to public health issues that do not provide short-term profitability be? Will this situation lead to long-term increases in the costs of health insurance, as is the case in some countries where the commercialisation and commodification of health care is a reality forged by historical tradition? All these questions need to be debated over the coming years in the field of the history of medicine because they are key issues for our welfare.

## Footnotes

1 An interesting reflection on the need to study hospitals can be found in Teresa Huguet-Termes et al., Ciudad y Hospital en el Occidente Europeo (1300–1700) (Lleida: Editorial Milenio, 2014), 13–24. For more on the increase in health insurance demand, see Melissa A. Thomasson, ‘From Sickness to Health: The Twentieth-Century Development of the Demand for Health Insurance’, *The Journal of Economic History*, 60, 2 (2000), 504–8.

2 More details on these aspects in Sarah Thomson and Elias Mossialos (eds), Private Health Insurance in the European Union (London: London School of Economics and Political Science, 2009).

3 Different forms of health care coverage took precedence in these countries depending on the period and the model adopted. The so-called mixed economy of welfare (coexistence of forms of solidarity, state action and private companies) was a preliminary step towards the creation of two basic models of health insurance by the mid-twentieth century; see Bernard Harris , The Origins of the British Welfare State: Social Welfare in England and Wales, 1800–1945 (Hampshire: Palgrave Macmillan, 2008). After the Second World War, state insurance prevailed in Western Europe, whereas private insurance companies took precedence in the United States. For more on these aspects, see Michael van der Linden (ed.), Social Security Mutualism: The comparative History of Mutual Benefit Societies (Bern: Peter Lang, 1996); Martin Gorsky , ‘The Growth and Distribution of English Friendly Societies in the Early Nineteenth Century’, The Economic History Review, 51, 3 (1998), 489–511 ; John E. Murray , Origins of American Health Insurance. A History of Industrial Sickness Funds (New Haven–London: Yale University Press, 2007); Michel Dreyfus , L*es assurances socials en Europe* (Rennes: Presses Universitaires de Rennes, 2009); David T. Beito , From Mutual Aid to the Welfare State. Fraternal Societies and Social Services, 1890–1967 (Chapel Hill: The University of North Carolina Press, 2020).

4 Information taken from Table 2.7 of the OECD Health Project 2004, *Private Health Insurance*; available at https://www.oecd.org/health/privatehealthinsuranceinoecdcountries-theoecdhealthproject.htm, 51. Definition of functions of private health insurance in box 2.2, 29. For more about health insurance in France, see Thomas Buchmueller and Agnes Couffinhal, ‘Private Health Insurance in France’, OECD Health Working Papers (Paris: OECD, 2012), 12; available at https://doi.org/10.1787/555485381821; in the UK, Yvonne Doyle and Adrian Bull , ‘Role of Private Sector in United Kingdom Health Care System’, The BMJ, 321, 7260 (2000)10.1136/bmj.321.7260.563PMC111844810968825, 563–5 and in Canada, Jeremiah Hurley and G. Emmanuel Guidon, *Private Health Insurance in Canada*, CHEPA Working Paper series, n. 08-04 (2008).

5 A compilation of studies on these typologies can be found in Bernard Harris and Paul Bridgen (eds), Charity and Mutual Aid in Europe and North America since 1800 (New York: Routledge, 2007).

6 The following works are noteworthy: Lindsay Granshaw and Roy Porter , The Hospital in History (London: Routledge, 1989); John Henderson , Peregrine Horden and Alessandro Pastore , ‘Introduction. The world of the hospital: Comparisons and continuities’, in John Henderson , Peregrine Horden and Alessandro Pastore (eds), The Impact of Hospitals 300-2000 (Bern: Peter Lang, 2007), 15–57 ; Gunter B. Risse , Mending Bodies, Saving Souls: A History of Hospitals (New York: Oxford University Press, 1999).

7 Huguet-Termes *et al.*, *op. cit.* (note 1). In the same vein, see also Christopher Bonfield, Jonathan Reinarz and Teresa Huguet-Termes, *Hospitals and Communities 1100-1960* (Oxford: Peter Lang, 2013). For Japan, see Pierre-Yves Donzé , ‘Hospital Construction and Planning in Japan: A Business between State Intervention and Free Competition (1918–1970)’. *Paper presented at the Workshop* Healthcare Industries in the Twentieth Century (Barcelona: University of Barcelona, 29 November 2016).

8 See Alfonso Herranz, ‘La difusión internacional de los seguros sociales antes de 1945’, in Jerònia Pons and Javier Silvestre (eds), *Los orígenes del estado de bienestar en España, 1900–1945.* (Zaragoza: Prensas Universitarias de Zaragoza, 2010), 51–83. In this section, it may also be useful to see the different case studies included in Martin Gorsky , Margarita Vilar-Rodríguez and Jerònia Pons-Pons (eds), The Political Economy of the Hospital in History (Huddersfield: University of Huddersfield Press; Open Access, 2020); available at https://unipress.hud.ac.uk/plugins/books/26/.

9 A term used from a more global perspective of welfare (coverage of a set of social risks, including sickness) by Harris and Bridgen, *op. cit.* (note 5).

10

Martin Gorsky and Sally Sheard (eds), Financing Medicine: The British Experience since 1750 (London: Routledge, 2006).

11 The main studies are as follows: For Germany, Sussane Hilger, ‘Welfare Policy in German Big Business after the First World War: Vereinigte Stahlwerke AG, 1926–33’, *Business History*, 40, 1 (1998), 50–76; Axel C. Hüntelmann, ‘Principles and Problems of Hospital Funding in Germany in the Twentieth Century’. in Martin Gorsky, Margarita Vilar-Rodríguez and Jerònia Pons-Pons (eds), *The Political Economy of the Hospital in History* (Huddersfield: University of Huddersfield Press, Open Access, 2020), 95–136. Paper presented at the I International Workshop *The Construction, Funding and Management of the Public and Private Hospital Systems of Developed Countries* (Seville, November 2017); for France, Jean-Paul Domin, *Une histoire économique de l’hôpital (XIXe-XXe siècles). Une analyse rétrospective du développement hospitalier, Tome I (1803–1945)* (Paris: Comité d’histoire de la Sécurité sociale, 2008); Christian Chevandier , L’hôpital dans la France du XXe siècle (Paris: Perrin, 2009) and for Britain, Steven Cherry, ‘Before the National Health Service: Financing the Voluntary Hospitals, 1900–1939’, *The Economic History Review*, 50 (1997), 305–26; Martin Gorsky , John Mohan and Martin Powell , ‘The Financial Health of Voluntary Hospitals in Interwar Britain’, Economic History Review, 55 (2002), 533–57; Barry Doyle , The Politics of Hospital Provision in Early Twentieth-Century Britain (London: Pickering and Chatto, 2014).10.3138/cbmh.34.2.52228920739

12

John Lapidus , The Quest for a Divided Welfare State: Sweden in the Era of Privatization (Cham: Palgrave Macmillan, 2019); John Lapidus , ‘Privatising, liberalising and dividing a welfare state without affecting universality? Debunking the myths surrounding the rapid rise of private health insurance in Sweden’, Health Economics, Policy, and Law, 17, 4 (2022), 367–79.3538291710.1017/S174413312200007X

13 Elias Mossialos *et al.* (eds), *Funding Health Care: Options for Europe* (Buckingham: Open University Press, 2002).

14

Paul Starr , Remedy and Reaction: The Peculiar American Struggle over Health Care Reform (New Haven: Yale University Press, 2011).

15

Jane Duckett , The Chinese State’s Retreat from Health: Policy and the Politics of Retrenchment (London: Routledge, 2011).

16

David de Ferranti , Paying for Health Services in Developing Countries: An Overview (World Bank Staff Working Papers; Washington DC: World Bank, 1985).

17 Moreover, it is interesting to see Christy F. Chapin , ‘The American Medical Association, Health Insurance Association of America, and Creation of the Corporate Health Care System’, Studies in American Political Development, 24, 2 (2010), 143–67; Christy F. Chapin , Ensuring America’s Health: The Public Creation of the Corporate Health Care System (New York: Cambridge University Press, 2015); Charles Rosenberg , The Care of Strangers. The Rise of America’s Hospital System (New York: Johns Hopkins University Press, 1987); Rosemary Stevens , In Sickness and in Wealth. American Hospitals in the Twentieth Century (New York: Johns Hopkins University Press, 1989).

18 Gorsky, Vilar-Rodríguez and Pons-Pons, *op. cit.* (note 8).

19

Christy F. Chapin , ‘Reply: What Historians of Medicine Can Learn from Historians of Capitalism’, Bulletin of the History of Medicine, 94, 3 (2020), 388–933341672310.1353/bhm.2020.0069; Chapin F. Chapin , ‘What Historians of Medicine Can Learn from Historians of Capitalism’, Bulletin of the History of Medicine, 94, 3 (2020), 319–673341671910.1353/bhm.2020.0065; Beatrix Hoffman , ‘Comment: What Historians of Medicine Can Learn from Historians of Capitalism’, Bulletin of the History of Medicine, 94, 3 (2020), 368–733341672010.1353/bhm.2020.0066; Nancy Tomes , ‘Comment: What Historians of Medicine Can Learn from Historians of Capitalism’, Bulletin of the History of Medicine, 94, 3 (2020), 373–8310.1353/bhm.2020.006733416721; Patrick Wallis , ‘Comment: What Historians of Medicine Can Learn from Historians of Capitalism’, Bulletin of the History of Medicine, 94, 3 (2020), 384–73341672210.1353/bhm.2020.0068; All of them participate in this debate.

20 Hoffman, *ibid.*

21 Chapin, *op. cit.* (note 19).

22

Karl Polanyi , The Great Transformation: The Political and Economic Origins of Our Time (New York: Rinehart and Farrar, 1944).

23 Hoffman, *op. cit.* (note 19).

24 Tomes, *op. cit.* (note 19).

25
*Ibid.*

26

Eli Ginzberg , ‘The Destabilization of Health Care’, The New England Journal of Medicine, 315, 18 (1986), 757–61352885110.1056/NEJM198609183151210.

27

Patrick Wallis and Mark S. R. Jenner (eds), Medicine and the Market in England and Its Colonies, c.1450–c.1850 (Basingstoke: Palgrave Macmillan, 2007).

28 Chapin, *op. cit.* (note 19).

29 For more details on this debate, see Chapin, *op. cit.* (note 19); Hoffman, *op. cit.* (note 19); Tomes, *op. cit.* (note 19); Wallis, *op. cit.* (note 19).

30

Josep Barceló-Prats , ‘Genealogía de la reforma hospitalaria en España: la gestación de una nueva cultura hospitalocéntrica de la sanidad’, Dynamis, 41, 1 (2021), 27–51 .

31 Jerònia Pons-Pons and Margarita Vilar-Rodríguez, *El Seguro de Salud Privado y Público en España: Su análisis en perspectiva histórica (1880–2010)* (Zaragoza, Prensas Universitarias de Zaragoza, 2014); Margarita Vilar-Rodríguez and Jerònia Pons-Pons (eds), *Un siglo de hospitales entre lo público y lo privado (1886–1986) [Financiación, gestión y construcción del sistema hospitalario español]* (Madrid: Marcial Pons, 2018). Compulsory sickness insurance was belatedly passed in Spain with serious financial limitations, in a country subject to severe political repression and hunger. See also Carlos Barciela , ‘Los años del hambre’, in Enrique Llopis and Jordi Maluquer (coords), España en crisis: las grandes depresiones económicas, 1348–2012 (Barcelona: Pasado y Presente, 2013), 165–92.

32 Margarita Vilar-Rodríguez and Jerònia Pons-Pons, ‘El debate en torno al seguro de salud público y privado en España: desde la transición política a la Ley General de Sanidad (1975–1986)’, *Historia y Política*, 39, 1 (2018), 261–90; Margarita Vilar-Rodríguez and Jerònia Pons-Pons, ‘Competition and Collaboration between Public and Private Sectors: The Historical Construction of the Spanish Hospital System (1942–1986)’, *Economic History Review*, 72, 4 (2019), 1127–636. For more on the evolution of hospital care in rural Spain, see Josep Barceló-Prats and Daniel Lanero-Táboas , ‘From Abandonment to Hospitalisation: Evolution of Hospital Care in Rural Spain (1939–1975)’, Social History of Medicine, 35, 2 (2022), 661–813555865210.1093/shm/hkab126PMC9086768.

33 There are three mutual funds for public employees: Muface, Mugeju and Isfas. Muface corresponds to civil servants, Mugeju corresponds to public employees in the judicial system and Isfas is for the state employees in the Armed Forces. For more on these special regimes, see Iván A. Rodríguez Caro , ‘El Régimen Especial de Seguridad Social de las Fuerzas Armadas: Una visión panorámica del mutualismo militar’, Revista del Ministerio de trabajo y asuntos sociales, 74 (2008), 35–56 .

34 Pons-Pons and Vilar-Rodríguez, *op. cit.* (note 31).

35
*Ibid.*, 329.

36

Margarita Vilar-Rodríguez and Jerònia Pons-Pons , ‘El papel de los hospitales municipales y provinciales en España desde una perspectiva histórica’, Dossier especial, Los factores condicionantes en la configuración histórica del sistema hospitalario en España, *Dynamis*
. Acta Hispanica ad Medicinae Scientiarumque Historiam Illustrandam, 41, 1 (2021), 79–110 .

37 The devolution of health care responsibilities to the autonomous communities and, consequently, the decentralisation of health policies in Spain, took place in various stages in a process that went from 1981 to 2002.

38 Vilar-Rodríguez and Pons-Pons, *op. cit.* (note 31).

39 Royal Decree 1088/1989, Boletín Oficial del Estado (official state gazette), 9 September 1989, no. 216, 28657–8. For more on these aspects, see also Sociedad Española de Salud Pública y Administración Sanitaria (SESPAS), *Informe SESPAS 1993 la salud y el sistema sanitario en España* (Barcelona: SG Editores, 1993), 191; Francisco Sevilla Pérez , ‘El cambio de un modelo mutual de Seguridad Social al Sistema Nacional de Salud II. El aseguramiento universal’, in Francisco Ortega and Fernando Lamata (eds), La década de la Reforma Sanitaria (Madrid: Exlibris Ediciones, 1996), 83–90 .

40

Roser Pérez Jiménez , ‘Políticas sanitarias y desigualdades en España’, in José Adelantado (coord.), Cambios en el Estado del Bienestar (Barcelona, Icaria, 2000), 251–84.

41 Francisco Comín Comín, ‘La fiscalidad del Estado del bienestar frente a la fiscalidad del franquismo (1940–2016)’, Comunicación presentada en La evolución de los sistemas fiscales desde la España medieval a la contemporánea: objetivos y consecuencias, Universidad de Málaga, 23 y 24 de junio, 2017; available at https://www.aehe.es/wp-content/uploads/2016/01/201709_Francisco-Comin.pdf; Francisco Comín Comín, ‘La transformación del Estado del Bienestar en España entre 1940 y 2018’, in Francisco Comín and Lluis Torró (eds), *El Estado del Bienestar en España: crisis económicas y desigualdad. Estudios en Homenaje a Salvador Salort* (Alicante: Publicaciones de la Universidad de Alicante, 2020), 43–75.

42

Sara Torregrosa , ‘Sistema fiscal y redistribución: la transición fiscal española (1960–1990)’, Perfiles Económicos, 1, 1 (2016), 149–80.

43 For further details, see José Luis Temes and Jesús Gil , El Sistema nacional de salud (Madrid: McGraw-Hill Interamericana, 1996); Pérez Jiménez, *op. cit.* (note 40).

44
*Boletín Oficial de Estado*, 26 April 1997, no. 100, 13449–50. Law 15/97 empowers the Government to create any entities of a public nature or public ownership allowed by law for the management and administration of health centres, which include, in the explanatory memorandum, foundations, consortiums and public companies.

45 Diario Médico digital archive ‘El IDIS, contra la decisión de romper conciertos con la privada’, 25 February 2016, 2.

46

Manuel Guerrero Castro , ‘El seguro de Asistencia Sanitaria y sus principales problemas’, Hacienda Pública Española, I (1986), 207–32.

47

UNESPA, Estadística del Seguro Privado 1984–1993 (Madrid: Servicio Actuarial, 1994), 251 .

48 Cinco Días, El País newspaper archive, ‘Hospitales en venta’, 13 November 2013; available at https://cincodias.elpais.com/cincodias/2013/11/13/empresas/1384363447_947237.html.

49 Law 15/97, of 25 April, passed by a majority of Parliament, consolidated the authorisation of new forms of management within the national health service, which entailed a change from the spirit of the *Ley General de Sanidad* of 1986, which had fundamentally based on the system on the direct management of the health care institutions of the Social Security.

50

Jesús M. González Pérez , ‘Mercado sanitario privado y territorio en Galicia. Neoliberalismo y nuevas pautas de comportamiento social’, Investigaciones Geográficas, 27 (2002), 205–26.

51 The HLA Hospital Group belongs to Asisa insurance company. It comprises seventeen hospitals and thirty-six multi-speciality medical centres; available at https://www.grupohla.com/es/about.

52 According to data from the IDIS report, *Análisis de la situación de la Sanidad Privada 2021*; available at https://www.fundacionidis.com/informes/analisis-de-situacion-de-la-sanidad-privada/sanidad-privada-aportando-valor-analisis-de-situacion-2021.

53 Obtained from Alimarket special report, *Grupos de Hospitales. El sector mantiene la tendencia a la concentración*, April and May (2021), 64.

54 Público digital archive, ‘Los hospitales de la Iglesia compiten con ventaja en la sanidad privada gracias a sus privilegios fiscales’; available at https://www.publico.es/sociedad/hospitales-iglesia-compiten-ventaja-sanidad-privada-gracias-privilegios-fiscales.html.

55 ‘Hospitales de la Iglesia, ¿sin ánimo de lucro?’, *Revista Médica*, 180, 13 April 2014; available at https://www.rmedica.es/edicion/180/hospitales-religiosos-sin-animo-de-lucro. In 2014, seven religious hospitals created the Hospitales Católicos de Madrid group, which has not stopped expanding in recent years.

56 Isanidad official website; available at https://isanidad.com/174068/13-grupos-hospitalarios-concentran-el-47-de-los-hospitales-privados/.

57 For a history of the group, see Cristina Cruz Serrano and Laura Jiménez Fernández, ‘Historia de un gigante hospitalario. ¿El fin de un legado familiar? “Grupo Quirón”’, *IE Business School*, GE1-140 (2017).

58 See the institutional website: https://dkvsalud.com/es/informacion-corporativa/historia.

59 El Confidencial digital archive; available at https://www.elconfidencial.com/economia/2012-03-22/los-hospitales-usp-y-quiron-se-fusionan-para-crear-un-gigante-de-la-sanidad-privada_417839/.

60 The resulting company would manage forty hospitals (including six university hospitals), over thirty health centres, six residential homes and the only health research institute accredited by the Carlos III Heath Institute. This group now provided more than 6 200 beds, employed 17 000 people and 8 000 collaborating doctors. El País newspaper archive, 17 June 2014.

61 La Voz de Asturias digital archive, ‘Así fraguó su fortuna Víctor Madera, el multimillonario médico ovetense’, 6 January 2017; available at https://www.lavozdeasturias.es/noticia/actualidad/2017/01/05/fraguo-fortuna-victor-madera-multimillonario-medico-ovetense/00031483640208944403581.htm.

62 The Spanish subsidiary of Capio, Capio Sanidad, managed more than twenty hospitals in Spain, spread out over five autonomous communities, including the Jiménez Díaz Foundation, Hospital Infanta Elena, Hospital de Sur and Hospital General de Catalunya. The resulting group had over one hundred thousand employees, ran 155 health centres, including forty-two hospitals and fifty-three health centres of another kind, and three hundred occupation risk prevention offices of the Quirónsalud network. Cinco Días, El País newspaper archive, 6 January 2011.

63 Cinco Días, El País newspaper archive, 18 June 2014. For the role of the executive Víctor Madera, see El País digital archive, ‘Víctor Madera, el amo de la sanidad privada en España’, 12 September 2016.

64 Archivo del Registro Mercantil de Madrid, inscripción 1, tomo 33894, hoja M-609942, October 2015. The company emerged from the acquisition by Quirón Hospitales, S.L. of the following companies: Idcsalud, S.L.; Ibérica de Diagnóstico y Cirugía, S.L.; Hospital Sur, S.L.; Salud Clínica del Pilar, S.L.; Centro FIV Recoletas, S.L.; Lesbas Directorship, S.L.; Grupo Hospitalarios Quirón, S.A., Quirón Bilbao, S.A.; Palace Helathcare, S.L., Teknon Healthcare, S.L.; Centro Médico Teknon, S.L.; Unidad Medicina Teknon, S.L., USP Mediplan Sport, S.L.; USP Araba Sport Clinic, S.L.; Clínica Maternal Nuestra Señora de la Esperanza, S.A.; USP Hospitales Internacionales, S.L. and DH Montserrat Spain, S.A.

65 Infolibre digital archive; available at https://www.infolibre.es/politica/gigante-aleman-fresenius-lidera-negocio-sanitario-espana-4-000-millones-tres-crecer-22-pandemia_1_1226270.html.

66 Archivo Registro Mercantil de Madrid, inscripción 80, tomo 38628, hoja M-609942, febrero del 2021.

67 La Voz de Asturias digital archive; available at https://www.lavozdeasturias.es/noticia/actualidad/2017/01/05/fraguo-fortuna-victor-madera-multimillonario-medico-ovetense/00031483640208944403581.htm.

68 For more information on the Gallardo family, see its institutional websites: https://gallardofamilygroup.com/es/origenes-e-historia/ and https://www.alimarket.es/sanidad/noticia/78566/goodgrower-notifica-a-competencia-la-compra-del-80--de-adeslas-hospitales.

69 Archivo del Registro Mercantil de Madrid, inscripción n. 1, tomo 1222, hoja M-214085, fs. 16–26, September 1966.

70 Archivo del Registro Mercantil de Madrid, inscripción n. 16, tomo 309, hoja 110 N, f. 35, June 1991. Unión Médica Regional sells to Adeslas, S.A. insurance company the portfolio of health care, sickness and accidents for the price of 58.8 million pesetas.

71 At this time, Adeslas, S.A. sold the shares of the following companies: Alianza Médica Leridana, S.A., Casa de Reposo y Sanatorio del Perpétuo Socorro, S.A.; Centro Médico Zamora, S.A., Clínica Parque de San Antonio, Sanatorio Nuestra Señora de la Salud, S.A., Sanatorio Virgen del Mar Cristóbal Castillo and Plazasalud. Archivo del Registro Mercantil de Madrid, inscripción n. 8, tomo 13222, hoja 214085 N, fs. 48–9, February 2004.

72 Expansión digital archive, ‘La Caixa pone a la venta los diez hospitales de Adeslas por 150 millones’, 11 February 2011; available at https://www.expansion.com/2011/02/11/empresas/banca/1297430115.html.

73 Archivo del Registro Mercantil de Madrid, inscripción 25, tomo 29542, hoja 214085, fs. 201–3, December 2012.

74 In March 2012, Goodgrower was represented by one of the three company administrators, José Luis Pardo Izquierdo, one of the company’s new directors. Archivo del Registro Mercantil de Madrid, inscripción 15, tomo 13222, hoja 214085 N, fs. 210–15, March 2012. The company lost its single shareholder status as the sole shareholder at this time (Caixabank, S.A.) sold 80% of the shares to Goodgrower, S.A. (f. 199).

75 Nisa was originally the owner of Hospital Valencia al Mar and Clínica Virgen del Consuelo. In 2020, it claimed thirty-one million pesetas from the Department of Economy and Finance of the Valencian regional government for health care services provided in 1997. El País newspaper archive, 10 January 2002.

76 Cinco Días, El País newspaper archive, 19 January 2017. This acquisition went ahead after a battle with a platform of Nisa shareholders (which represented 53% of the shareholders) that opposed the sale.

77 El País newspaper archive, 22 October 2003.

78 Cinco Días, El País newspaper archive, 4 April 2005.

79 ABC newspaper archive, 11 September 2007.

80 ABC newspaper archive, 16 November 2017.

81 Expansión digital archive 12 January 2018, ‘Quienes son los dueños de los Hospitales en España’; available at https://www.expansion.com/empresas/2018/01/12/5a57d5b946163f045e8b460e.html.

82 El Español digital archive; available at https://www.elespanol.com/invertia/observatorios/sanidad/20210113/gallardo-quedan-vithas-comprar-parte-criteriacaixa/550945591_0.html.

83

Marisol Rodríguez , ‘El sector público y el sector privado de la sanidad: ¿estabilidad o cambio?’ Gaceta Sanitaria, 33, 6 (2019), 499–501 3158577110.1016/j.gaceta.2019.08.001.

84 A foundation set up in May 2010, which brings together the private health sector and has as its goal, according to its website: ‘promote an improvement in health of the population and defend the Spanish health system as a whole, by means of developing the potential of the contribution of private health care’. Its board is made up of the most important private hospital companies and insurance companies: Aces (Associació Catalana d’Entitats de Salut), Adeslas, Analiza, Asisa, Axa, Caser seguros, Catalana Occidente, Divina seguros, DVK salud, Farma industria, Tecnología sanitaria, Genesis Care, Recoletas, Red Hospitalaria, Hospitales Católicos de Madrid, HM Hospitales, Hospiten, IMQ, Mapfre, Orden Hospitalaria de San Juan de Dios, Hospital Perpetuo Socorro, Quirón Salud, Ribera, Sanitas (BUPA), Teladoc Health, Viamed and Vithas; available at https://www.fundacionidis.com/miembros/patronos (consulted on June 2022).

85 ‘El IDIS, contra la decisión de romper conciertos con la privada’, *Diario Médico*, 25 February 2016, 2.

86 Italy is a similar case, see Daniela Felsini and Fernando Salsano ‘Le imprese private nel sistema sanitario italiano’, in Margarita Vilar and Jerònia Pons (eds), Los hospitales y las pandemias en España e Italia desde una perspectiva histórica (Alicante: Pub. Universitat d’Alacant, 2022), 385–410 .

